# Challenges in scaling up a perfusion process

**DOI:** 10.1186/1753-6561-5-S8-P122

**Published:** 2011-11-22

**Authors:** Vana Raja, Saravanan Desan, Ankur Bhatnagar, Anuj Goel, Harish Iyer

**Affiliations:** 1 Cell Culture Lab, Biocon Limited, Bangalore, India

## Introduction

Perfusion process involves retention of the cells inside the bioreactor while simultaneously removing spent medium and adding fresh medium continuously. The flow rate of addition of fresh and removal of spent medium are generally kept the same (perfusion rate) to maintain the culture volume inside the bioreactor. Cell retention is possible with many devices but the reliability and consistency in performance of these devices remains a major challenge for design, operation and scale-up of perfusion processes. For a filtration based retention device, successful scale up depends on cell retention efficiency, prevention of filter fouling and the similarity of perfusion equipment between small and large scale. We present a case study where a perfusion process from a 2L (Liter) bioreactor is scaled up to manufacturing scale of 1KL (Kilo Litre).

## Materials and methods

A NS0 host cell line cultured in protein free medium was used. At lab scale 2L stirred tank bioreactors with internal spin filters as the retention device were used. The 1KL production bioreactor used closed external rotating filters for cell retention. Cell count and viability were determined using Hemocytometer and Trypan Blue dye exclusion. Glucose and lactate were measured using YSI 2700 analyzer and product concentration by Affinity chromatography.

## Results and discussions

### Development of perfusion process and scale up

A perfusion process was developed which consisted of a batch phase for cell growth followed by perfusion phase once the cell density reaches the desired level. This process was scaled up by scaling up the scale dependent parameters linearly same while maintaining the scale independent factors within the acceptable range. The filter parameters such as mesh type and filter area per unit bioreactor volume were however not comparable between the scales.

### PB-1 (Production Batch no. 1)

The growth rate during the batch phase of the run was comparable to the lab scale. However, during the perfusion phase, the maximum cell density was observed to be only about 25% of the lab scale. Due to the lower cell counts, the perfusion rates were also proportionally reduced. Together this resulted in obtaining only ~25% of the expected product yield.

As part of investigation, the following factors were analyzed:

a) Inoculum and “medium lot” used in PB-1 – A control batch was run in the lab with the same inoculum and medium as used in the PB-1 run. This batch showed normal lab batch profiles ruling out these factors as possible cause for the underperformance of the production batch.

b) Pumps used for perfusion – The lab scale used peristaltic pumps while in manufacturing pulsating pumps were used. These pulsating pumps could influence cell retention and hence were replaced with peristaltic pumps for future production batches.

### PB-2 (Production Batch no. 2)

Changing the pump type resulted in better cell retention. However, soon the retention started dropping, indicating cell loss from the bioreactor. Visual inspection of the filter mesh (mesh Type A) showed significant mesh deformation. This could have resulted due to the fragile nature of the mesh which could not withstand the negative pressure generated inside the closed filter housing due to suction forces and filter fouling. The mesh Type A was also found to be fouling very fast due to it mesh weave design. Two other mesh types (B & C) with different mesh weave patterns and mechanical strengths were evaluated. Type B showed poor cell retention due to bigger pore size. Type C showed better cell retention and also did not deform easily during filter fouling. Thus the filter mesh was changed from Type A to Type C in the production system.

### PB-3 (Production Batch no. 3)

**Figure 1:**
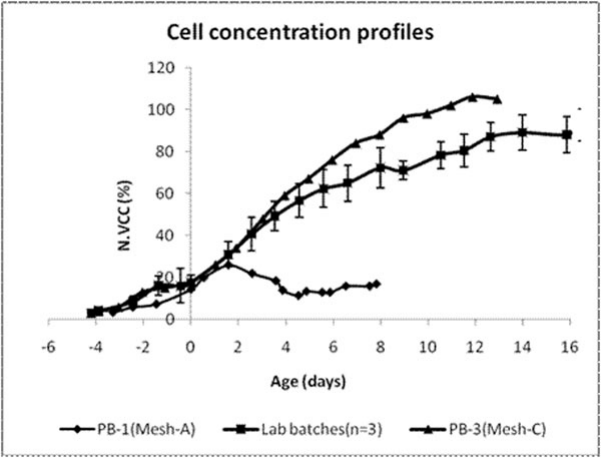
Cell count profiles from the lab batches and the production batches with different mesh types.

Note: N. VCC – normalized viable cell concentration

The filter with mesh C showed cell retention better than even the lab scale. This resulted in achieving cell densities higher than the range of the lab batches (Figure 1). The perfusion rates were increased by about 20% from the lab scale to address the nutritional requirements of the higher cell numbers. The product yield was also proportionally higher by ~20%.

### Product quality analysis

Analysis showed that the product obtained from batch PB-3 was significantly different in quality (charge distribution) compared to product from PB-1,2 and lab batches. The following were evaluated as possible reasons for the observed differences:

a) High perfusion rates compared to lab and PB-1,2 batches resulting in shorter product residence time inside the bioreactor.

b) Different lactate and pCO2 levels maintained (due to higher perfusion rates) which also resulted in different pH profiles.

### PB-4 (Production Batch no. 4)

Batch PB-4 was run with perfusion flow rates comparable to the lab batches. The additional nutritional requirement for the higher cell concentration was addressed by making the fresh medium more concentrated. pH was also controlled using the CO_2_ and base combination. The cell concentration obtained was similar to PB-3. Lowering the perfusion flow rates also helped in delaying the clogging of the filter.

Product analysis showed that the changes done in the batch helped in bringing the product quality closer to (within acceptable range) the lab batches. The results are shown in the Table 1. Two differently charged species Type-1 and Type-2 are used for comparison.

**Table 1:** Product quality comparison of PB-3 and PB-4 batches.

Perfusion lots	PB-3		PB-4	
	Type-1*	Type-2*	Type-1*	Type-2*

1	112	74	59	139
2	47	184	66	126
3	43	195	73	116
4	41	198	73	120
5	39	208	82	109
6	39	209	82	118

* % of Lab batch profiles.

## Summary

Development and scale-up of a perfusion process has challenges due to the complex nature of the process and unavailability of direct scale-up of the perfusion equipment. The initial scale-up to production scale resulted in poor cell growth profile. Upon investigation, the reason for low cell concentration was attributed to poor cell retention by the perfusion device. Changes were introduced in the type of mesh used for filter construction and perfusion pumps to improve retention. These modifications helped in better cell culture profiles and yields. However, the product quality was impacted because of these changes. Further changes in the perfusion flow rates were done to address the product quality differences.

